# The impact of sMICA/sMICB on immunochemotherapy outcomes in newly diagnosed diffuse large B-cell lymphoma

**DOI:** 10.3389/fonc.2023.1194315

**Published:** 2023-11-16

**Authors:** Sang Eun Yoon, Sujin Park, Junhun Cho, Kyung Ju Ryu, Booma Yandava, Sewon Lee, Seok Jin Kim, Won Seog Kim

**Affiliations:** ^1^ Division of Hematology-Oncology, Department of Medicine, Samsung Medical Center, Sungkyunkwan University School of Medicine, Seoul, Republic of Korea; ^2^ Department of Pathology, Samsung Medical Center, Sungkyunkwan University School of Medicine, Seoul, Republic of Korea; ^3^ Department of Health Sciences and Technology, Samsung Advanced Institute for Health Sciences and Technology, Sungkyunkwan University, Seoul, Republic of Korea; ^4^ Samyang Biopharm USA, Cambridge, MA, United States; ^5^ Division of Hematology-Oncology, Department of Internal Medicine, Ewha Womans University Mokdong Hospital, Seoul, Republic of Korea

**Keywords:** tumor microenvironment, natural killer group 2 member D, MHC class I-related chain A, MHC class I-related chain B, newly diagnosed diffuse large B-cell lymphoma

## Abstract

**Introduction:**

Soluble MHC class I-related chain A (sMICA) and B (sMICB) play a critical role tumor evolution and poor prognosis through an immune evasion mechanism. Thus, this study determines the interaction between sMICA/sMICB and the tumor immune environment in newly diagnosed diffuse large B-cell lymphoma (ND-DLBCL).

**Methods:**

We analyzed sMICA/sMICB, cytokine in serum, and macrophage polarization analysis in tissue samples before the first chemotherapy administration. This research was performed to investigate the correlation between sMICA/sMICB expression and treatment outcomes as well as their influence on the immune system within ND-DLBCL.

**Results:**

Of the 262 patients, 47.3% (n = 124) presented stage III or IV at diagnosis and 50.8% (n = 133) had a high International Prognostic Index (IPI ≥ 3). The patients with high (p = 0.034 and 0.004), elevated lactate dehydrogenase (p = 0.002 and 0.030), advanced stage (p = 0.003 and 0.012), and higher IPI risk (p = 0.009, and 0.032) correlated with the detection of sMICA or sMICB. The median progression-free survival (PFS) of patients with sMICA (p = 0.006) or sMICB (p =0.032) was inferior. Among the patients with advanced-stage or high IPI, those with sMICA or sMICB presented an inferior PFS and OS compared to those without. TNF-a, a pro-inflammatory cytokine, showed statistical significance with detected sMICA (p = 0.035) or sMICB (p = 0.044). Among anti-inflammatory cytokines, IL-1RA (P-value = 0.013) and IL-10 (p = 0.005) were associated with detecting sMICB, but not sMICA. In tissue samples, sMICA or sMICB detection did not correlate with the CD68/CD163 ratio.

**Discussion:**

Conclusively, the identification of sMICA/sMICB presented unfavorable immunochemotherapy outcomes, and it was assumed that sMICA or sMICB and various cytokines interact, but the relationship with macrophage differentiation is unclear. Therefore, further research is needed to determine the relationship between sMICA/sMICB and tumor microenvironment in DLBCL.

## Introduction

Natural killer group 2 member D (NKG2D) is an activating transmembrane receptor found on natural killer (NK) cells, CD8+ cytotoxic T cells, and macrophages (1). NKG2D plays a vital role in recognizing target cells and induces cell lysis through the cytotoxic functions in the cellular immune surveillance pathway. NKG2D ligands (class I MHC-like proteins, including MHC class I-related chain A (MICA) and MICB) are mainly present in tumor cells, and not normal cells (2). Therefore, under immune surveillance, the linkage of NKG2D and NKG2DL in tumor cells activates transcriptional upregulation related to cellular or genomic stress and eventually leads to tumor cell lysis. Unfortunately, tumor cells have various mechanisms to evade immune surveillance, and one of them is cleavage of MICA/MICB from the cell membrane using surface proteases (3). Therefore, previous research has found that higher concentrations of soluble MICA (sMICA) and sMICB molecules in serum play a critical role in tumor evolution and poor prognosis through an immune evasion mechanism (4, 5). However, few cases have reported the contribution of sMICA/sMICB in the development or acceleration of diffuse large B-cell lymphoma (DLBCL) (6). Thus, the role of sMICA/sMICB in DLBCL is still unclear.

Various cytokines play critical roles in the etiology of lymphoid malignancies, which arise from innate or adaptive immunity during different maturation periods of NK, T, and B lymphocytes (7, 8). Interestingly, it was reported that the tumor-derived sMICA and sMICB seemed to contribute to the upregulation of pro-inflammatory cytokines such as interleukin (IL)-2, IL-12, IL-18, IL-15, and interferon (IFN)-γ to impair T-cell activation (9, 10). In addition, sMICA/sMICB could mobilize the tumor microenvironment to discourage innate immunity against various antigens and abnormal cells as a part of upfront immune surveillance (11). According to a previous study, patients with higher serum levels of sMICA and sMICB might diagnose in an advanced stage (12). Therefore, it could be assumed that higher sMICA and sMICB would coordinate cell signaling pathways between normal immune cells and cancer cells by inducing pro-inflammatory cytokine secretion to promote a tumor-friendly environment.

Additionally, tumor-associated macrophages (TAMs) are associated with tumor proliferation, invasion, angiogenesis, metastasis, and suppression of anti-tumor immunity as intrinsic cellular components. Especially, DLBCL with M1-polarization (high CD68/CD163 ratio) described by positive CD68 immunohistochemistry staining presented better treatment response and survival outcomes than M2-polarization (low CD68/CD163 ratio) (13, 14). It is known that cytokines contribute to macrophage polarization, but the role of sMICA and sMICB in these complex interactions is not yet known (15).

Although the primary etiology of DLBCL is the result of accumulated genetic mutations in proto-oncogenes and tumor suppressor genes, the microenvironment of DLBCL is not clearly understood and is expected to affect therapeutic responses to immunochemotherapy and R-CHOP (rituximab, cyclophosphamide, doxorubicin, and prednisolone). Thus, targeting sMICA/sMICB could reveal the reasons for the immune escape of tumor cells and the mechanisms of immunochemotherapy resistance. Therefore, we conducted the study to demonstrate the influence of sMICA/sMICB on the immune escape of newly diagnosed DLBCL (ND-DLBCL). In addition, we discuss the role of sMICA/sMICB in the tumor immune environment through a comprehensive analysis of sMICA/sMICB, cytokine, and macrophage polarization.

## Methods

### Study information

**Figure 1:**
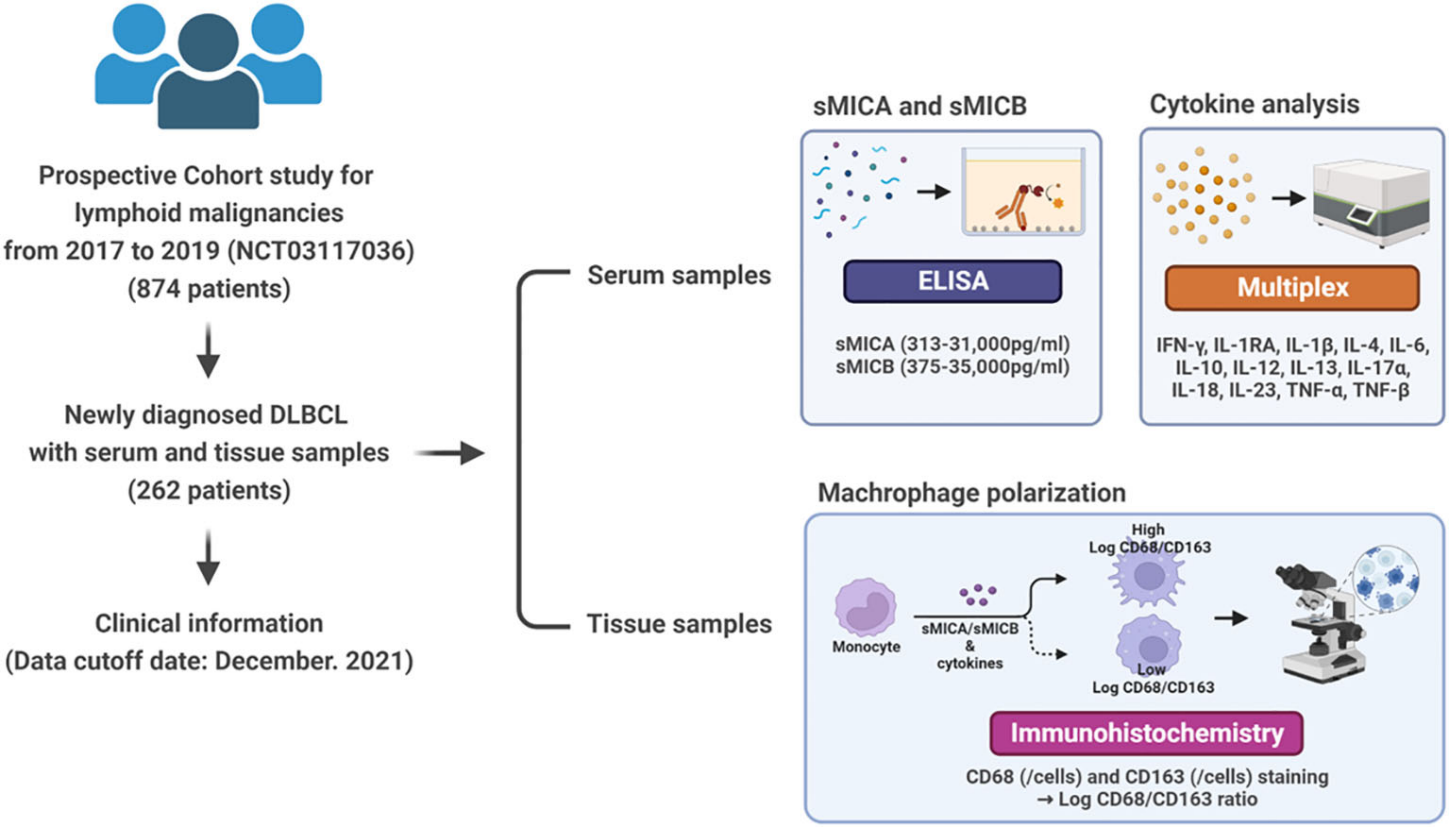
Study overview from sample collection to analysis of sMICA/sMICB, cytokines, and macrophage polarization.

We conducted the study to comprehensively analyze the tumor immune microenvironment interpreted by sMICA and sMICB in patients with newly diagnosed DLBCL. Our institution has been prospectively conducting a lymphoid malignancy cohort and collecting serum, peripheral blood mononuclear cells, and tissue samples from all enrolled patients at diagnosis or relapse/refractory timing after obtaining patient consent. The study population was enrolled in patients diagnosed with ND-DLBCL who had serum samples and survival outcomes from prospective cohort studies between September 2017 and December 2019 (NCT03117036). Therefore, through assessing the clinical information and sample storage status, a total of 262 patients who were newly diagnosed with DLBCL (ND-DLBCL) were enrolled in this study (**Figure 1**).

We collected the pretreatment characteristics of the patients and serum samples to determine sMICA/sMICB levels and cytokine analysis before the first administration of chemotherapy. The clinical information searched included gender, age, Eastern Cooperative Oncology Group (ECOG) performance status, presence of B-symptoms, complete blood count, serum lactate dehydrogenase (LDH) and β-2 microglobulin (B2M) concentrations, bone marrow involvement, International Prognostic Index (IPI), and Ann Arbor stage. Additionally, the cell-of-origin (COO) of DLBCL was demonstrated at the time of diagnosis by a lymphoma pathologist (J.H.). The Hans algorithm classified DLBCL into germinal centre origin (GCB) and non-GCB subtype subgroups. All patients were treated with R-CHOP chemotherapy for about 4 to 6 cycles, and the therapeutic response was evaluated every three cycles according to the Lugano response criteria (16). The cut-off date for this study was December 2021. This study was approved by the Samsung Medical Center IRB and was performed under the relevant guidelines and regulations, including the Declaration of Helsinki.

### Analysis of sMICA/sMICB

The level of sMICA/sMICB from serum was analyzed quantitatively by ELISA using R&D Systems™ DuoSet® ELISA kit for human MICA/MICB. The assay was conducted in compliance with the current industry practices. Assay specification of sMICA and sMICB demonstrated a range 313-31,000 pg/ml and 375-35,000pg/mL. The patients were distributed into low and high-detection groups based on the median value of sMICA and sMICB levels in the serum for further data analysis.

### Analysis of cytokines

We used serum samples collected at diagnosis and stored at −80°C until analysis to measure serum cytokines. Pro-inflammatory cytokines (IFN-γ, IL-1β, IL-6, IL-12, IL-17α, IL-18, IL-23, and TNF-α) and anti-inflammatory (IL-1RA, IL-4, IL-10, and IL-13) were measured with a Procarta cytokine profiling kit (Panomics, Fremont, CA, USA) using the Bio-Plex Cytokine Assay System (Bio-Rad Laboratories, Hercules, CA, USA) according to the manufacturer’s instructions (17, 18). Optimal cut-off values for cytokines were determined by whether the value could discriminate clinical outcomes such as negative or positive detection.

### Analysis of macrophage polarization from tissue samples

Formalin-fixed paraffin-embedded (FFPE) blocks were retrieved from the archive of the department of pathology, Samsung Medical Center, and were cut into 4-μm-thick slices (**Supplemental Figure 1**). The Opal Polaris 7-color automation IHC kit (NEL871001KT, AKOYA) was used with a Leica BOND Rx autostainer. All FFPE slides were deparaffinized and rehydrated, and heat-induced epitope retrieval was performed in ER1 (citrate based, pH 6) solution heated at 98°C for 20 min for CD68 (clone 514H12, Ventana), and in ER2 (EDTA based, pH9) solution heated at 98°C for 20 min for CD163 (clone EPR19518, Abcam), and CD20 (clone L26, Dako). All slides were treated with blocking buffer (Akoya) and then incubated with primary antibody for 30 minutes, followed by incubation with HRP-conjugated secondary antibodies (Ms+Rb polymer, Akoya). For CD68 and CD163, slides were subsequently incubated with Opal dyes and amplified at 620, 520, and 480 nm. For CD20, slides were incubated with TSA Plus DIG (Akoya) before they were incubated with Opal dye and amplified at 780 nm. All slides were rinsed with wash buffer (BOND wash sol 10×, Leica) at each step. For the final step, all slides were mounted with ProLong Gold AntiFade Mountant with DAPI for nuclear staining (Invitrogen).

All fluorescently labeled slides were scanned on a Vectra Polaris (Akoya, Menlo Park, CA, USA) at 20× magnification with appropriate exposure times. Using a Phenochart Whole Slide Viewer (Akoya, Menlo Park, CA, USA), two representative fields (1.28 mm^2^) with average tumor density from the tumor area of each case were selected and annotated for 72 cases. InForm 2.8 software (Akoya, Menlo Park, CA, USA) was used for quantitative image analysis. In this study, algorithms were trained to phenotype tumor cells as positive or negative for each antibody using ten selected images and then applied to all remaining 135 images. Total cell counts, positive cell counts for each antibody, positive cell percentage, and positive cell density were calculated using phenoptrReports (Akoya, Menlo Park, CA, USA).

### Statistical analysis

Descriptive statistics were determined as proportions and medians. The intergroup comparisons for categorical variables were assessed by Fisher’s exact test. An independent T-test was performed to compare the two groups of continuous variables. In the comparison of all variables, P-value was considered statistically significant at less than 0.05. Progression-free survival (PFS) was calculated as the date from diagnosis to disease progression or death related to any cause. Overall survival (OS) was defined as the period from the date of diagnosis to death or the last date of follow-up. Survival curves were described using Kaplan–Meier estimates and were compared between groups using the log-rank test. The Cox proportional hazards regression model was administered for univariate and multivariate analyses. Statistical analyses were performed using an IBM PASW version 25.0 software program (IBM SPSS Inc., Armonk, NY, USA).

## Results

### Baseline characteristics according to sMICA and sMICB detection

Of the 262 patients, slightly more were male (n = 145, 55.3%) than female (n = 117, 44.7%). Moreover, the number of patients over 60 was 125 (47.7%), and most patients (n = 256, 97.7%) presented a good general condition (ECOG PS 0-1). Elevated B2M and LDH levels were demonstrated in 34.1% (n = 86) and 40.5% (n = 106) of patients at diagnosis. Among the 228 patients whose COO data were available, 147 patients (56.1%) were classified as having DLBCL with non-GCB type, and 81 patients (30.9%) were allocated as DLBCL with GCB type. Of 214 patients (81.7%) with extranodal involvement, 29 (11.1%) presented central nervous system (CNS) involvement, 47.3% of patients (n = 124) presented stage III or IV at the diagnosis, and higher IPI (≥ 3) occurred in 133 patients (50.8%). Among 262 patients, sMICA and sMICB were analyzed in 260 and 262 cases, respectively. Fifty-three (n=53/262, 20.2%) presented both sMICA and sMICB, 121 (n=121, 46.2%) showed a single marker between sMICA or sMICB, and 88 (n=88/262, 33.6%) did not demonstrate sMICA and sMICB. Overall, 122 (n = 122/260, 46.9%) patients were found to have sMICA and 105 (n = 105/262, 40.1%) were found to have sMICB. According to a comparison between patients with or without sMICA and sMICB, those with high B2M (p = 0.034 and 0.004), elevated LDH (p = 0.002 and 0.030), advanced stage (p = 0.003, and 0.012) and higher IPI risk (p = 0.009, and 0.032) had a statistical correlation with the detection of sMICA or sMICB in serum (**Table 1**).

**Table 1:** Baseline clinical characteristics and comparison according to detection of sMICA and sMICB .

Variables		Total (N=262)		sMICA (N=260)					sMICB (N=262)				
				Not Detected (n = 138)		Detected (n = 122)		P	Not detected (n = 157)		Detected (n = 105)		P
		N	%	N	%	N	%		N	%	N	%	
Gender	Male	145	55.3	75	52.1	69	47.9	0.803	84	57.9	61	42.1	0.526
	Female	117	44.7	63	54.3	53	45.7		73	62.4	44	37.6	
Age	< 60 years	137	52.3	73	53.7	63	46.3	0.901	80	58.4	57	41.6	0.616
	≥ 60 years	125	47.7	65	52.4	59	47.6		77	61.6	48	38.4	
ECOG	PS 0-1	256	97.7	135	53.1	119	46.9	1.000	154	60.2	102	39.8	0.686
	PS 2-4	6	2.3	3	50.0	3	50.0		3	50.0	3	50.0	
B-SX	Absence	236	90.1	128	54.7	106	45.3	0.147	137	58.1	99	41.9	0.090
	Presence	26	9.9	10	38.5	16	61.5		20	76.9	6	23.1	
B2M*	Normal	166	65.9	94	57.3	70	42.7	0.034	111	66.9	55	33.1	0.004
	Elevated	86	34.1	37	43.0	49	57.0		41	47.7	45	52.3	
LDH	Normal	156	59.5	95	60.9	61	39.1	0.002	102	65.4	54	34.6	0.030
	Elevated	106	40.5	43	41.3	61	58.7		55	51.9	51	48.1	
EBV	Not detected			123	89.1	110	90.2	0.841	148	94.3	87	82.9	0.004
	Detected			15	10.9	12	9.8		9	5.7	18	17.1	
COO	Non-GCB	147	56.1	73	50.3	72	49.7	0.248	92	62.6	55	37.4	0.087
	GCB	81	30.9	49	60.5	32	39.5		41	50.6	40	49.4	
	NOS	34	13.0	16	47.1	18	52.9		24	70.6	10	29.4	
Extranodal	Involved	214	18.3	110	51.9	102	41.7	0.429	129	60.3	85	39.7	0.871
	Not involved	48	81.7	28	58.3	20	48.1		28	58.3	20	41.7	
CNS	Involved	233	88.9	130	56.0	102	44.0	0.005	142	60.9	91	39.1	0.422
	Not involved	29	11.1	8	28.6	20	71.4		15	51.7	14	48.3	
Stage	I/II	138	52.7	85	62.0	52	38.0	0.003	93	67.4	45	32.6	0.012
	III/IV	124	47.3	53	43.1	70	56.9		64	51.6	60	48.4	
IPI	0-1	129	49.2	79	61.2	50	38.8	0.009	86	66.7	43	33.3	0.032
	≥2	133	50.8	59	45.0	72	55.0		71	53.4	62	46.6	

sMICA, soluble major histocompatibility complex class I-related chain A; sMICB, soluble major histocompatibility complex class I-related chain B; P, P-value; ECOG, European Cooperative Oncology Group; PS, Performance status; LDH, Lactate Dehydrogenase; EBV, Epstein Barr virus; COO, Cell-of-origin; IPI, International prognostic index.

*Evaluable patient number of B2M was 250 among 260 or 262 patients, and the number of evaluable patients was 119 for sMICA or sMICB detection.

### The relationship with response rate and survival of sMICA and sMICB

**Figure 2:**
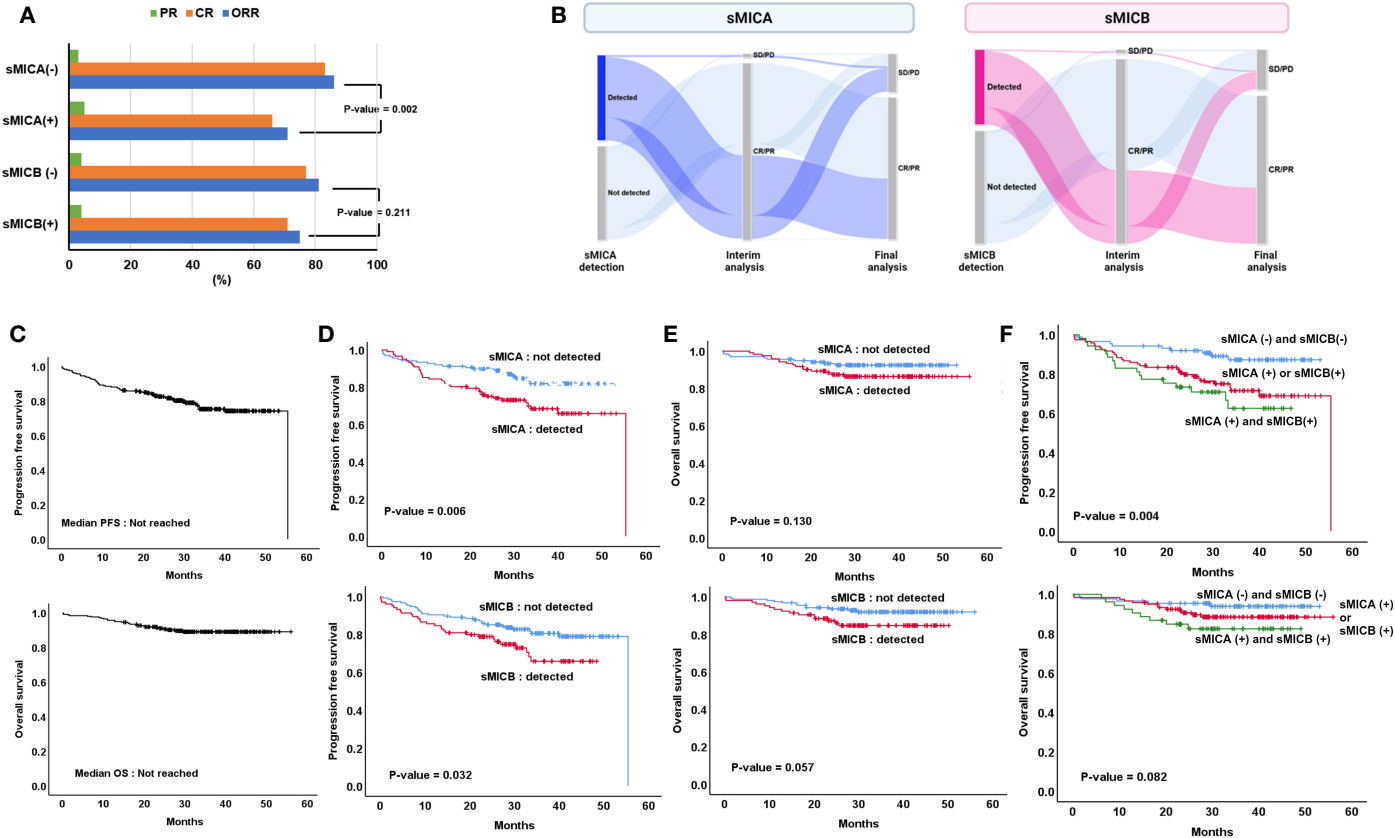
Treatment outcome according to the detection of sMICA or sMICB **(A)** Changes in interim and final treatment response **(B)**, overall progression-free survival (PFS) and overall survival (OS) **(C)**, comparison of PFS **(D)**, and OS **(E)** according to the detection of sMICA or sMICB, Assessment of PFS and OS according to (1) sMICA (-) & sMICB (-), (2) sMICA (+) or sMICB (+) and (3) sMICA (+) & sMICB (+) **(F)**.

All patients received R-CHOP as a front-line treatment, and the best response rate was 97.3% (241 CR, 12 PR, 1 SD, 4 PD) among 260 patients who were available to undergo response evaluation. Moreover, among 258, the final response rate was 78.7% (n=203/258), and 54 patients experienced disease progression at the cut-off date. The therapeutic response rate was superior in the patients who did not have sMICA (n=116/138, 86.6%) and inferior in those who had sMICA in serum (n = 86/122, 70.5%, p = 0.002). However, a significant comparison of therapeutic response rate was not shown according to the presence or absence of sMICB (p = 0.218, **Figure 2A**). In addition, the patients in who sMICA and sMICB were detected experienced a more frequent transformation from interim assessment CR/PR to final assessment SD/PD than those who did not have sMICA/sMICB in serum (**Figure 2B**).

During the 34.7 (95% CI 32.0-37.4) months median follow-up period, the PFS and OS were not reached at the median values (**Figure 2C**). Inferior PFS was found in patients who had sMICA than those who did not detect sMICA (not reached the median value [NR] vs. 55.4 months, p = 0.006, **Figure 2D**). Moreover, the patients who presented sMICB demonstrated poor PFS compared to those who did not present sMICB (NR vs. 55.4 months, p = 0.032, **Figure 2D**). However, the median OS was statistically similar in both patient groups, whether or not sMICA (NR vs. NR, p = 0.130) or sMICB (NR vs. NR, p = 0.057) were detected (**Figure 2E**). Additionally, the patients who detected both sMICA and sMICB showed poorer PFS compared to those who did not detect both sMICA and sMICB or only one positive out of two (p=0.004, **Figure 2F**). However, statistical significance was not proven for OS (p=0.082).

**Figure 3:**
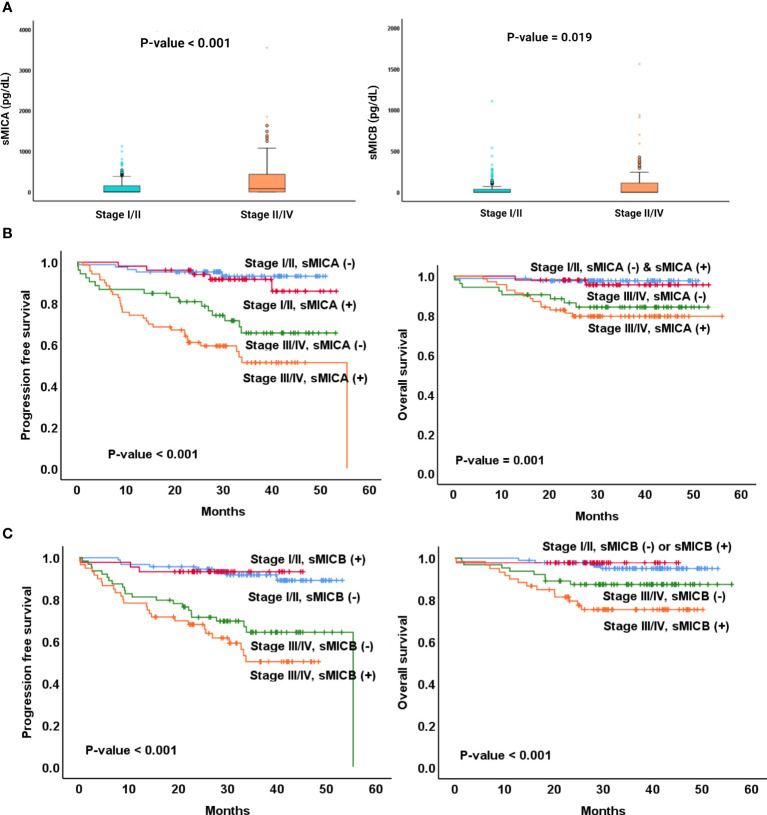
Comparison of median sMICA/sMICB levels between stage I/II or III/IV **(A)** Comparison of PFS and OS among four different categories: (1) the patients with stage I/II and sMICA not detected, (2) those with stage I/II and sMICA detection, (3) the patients with stage III/IV and sMICA not detected, and (4) those with stage III/IV and sMICA detection **(B)**, comparison of PFS and OS among four different categories: (1) the patients with stage I/II and sMICB not detected, (2) those with stage I/II and sMICB detection, (3) the patients with stage III/IV and sMICB not detected, and (4) those with stage III/IV and sMICB detection **(C)**.

We next performed subgroup analyses according to the stage (stage I/II versus stage III/IV). The median value of sMICA was higher in patients with advanced stages than in a limited stage (336.5 pg/dL, range 9.9-3554.5 vs. 292.2 pg/dL, range 12.5-1125.4, p < 0.001). Moreover, the advanced-stage patients presented elevated median sMICB than limited-stage patients (123.0 pg/dL, 0.0-1100.0 vs. 119.5 pg/dL, range 5.2-1560, p = 0.019, **Figure 3A**). In addition, the patients diagnosed with advanced stage showed a poorer PFS (p < 0.001) and OS (p < 0.001) than those with a limited stage (**Supplementary Figure 2A**). The stage III or IV patients who had sMICA or sMICB presented an inferior survival outcome in terms of PFS and OS compared to the same stage patients without sMICA or sMICB (**Figures 3B, C**).

According to the comparison between IPI 0-1 (Low) versus IPI ≥ 2 (High), the median value of sMICA (0.0 pg/dL, range 0.0-1125.4 vs. 48.9pg/dL, range 0.0-3554.5, p < 0.001) was higher in patients with IPI ≥ 2 than in those with IPI 0-1 (**Supplementary Figure 2B**). However, there was no difference in the median value of sMICB regardless of low or high IPI (0.0 pg/dL, 0.0-1100.0 vs. 0.0 pg/dL, range 0.0-1560.0, p = 0.074, **Supplementary Figure 2B**). In addition, the patients with estimated high IPI presented poorer PFS (p < 0.001) and OS (p < 0.001) than those with a limited stage (**Supplementary Figure 2C**). Among the patients with the same IPI status, those who had sMICA or sMICB presented an inferior PFS and OS than those who did not (**Supplementary Figures 2D, E**).

### Serum cytokine and survival

**Figure 4:**
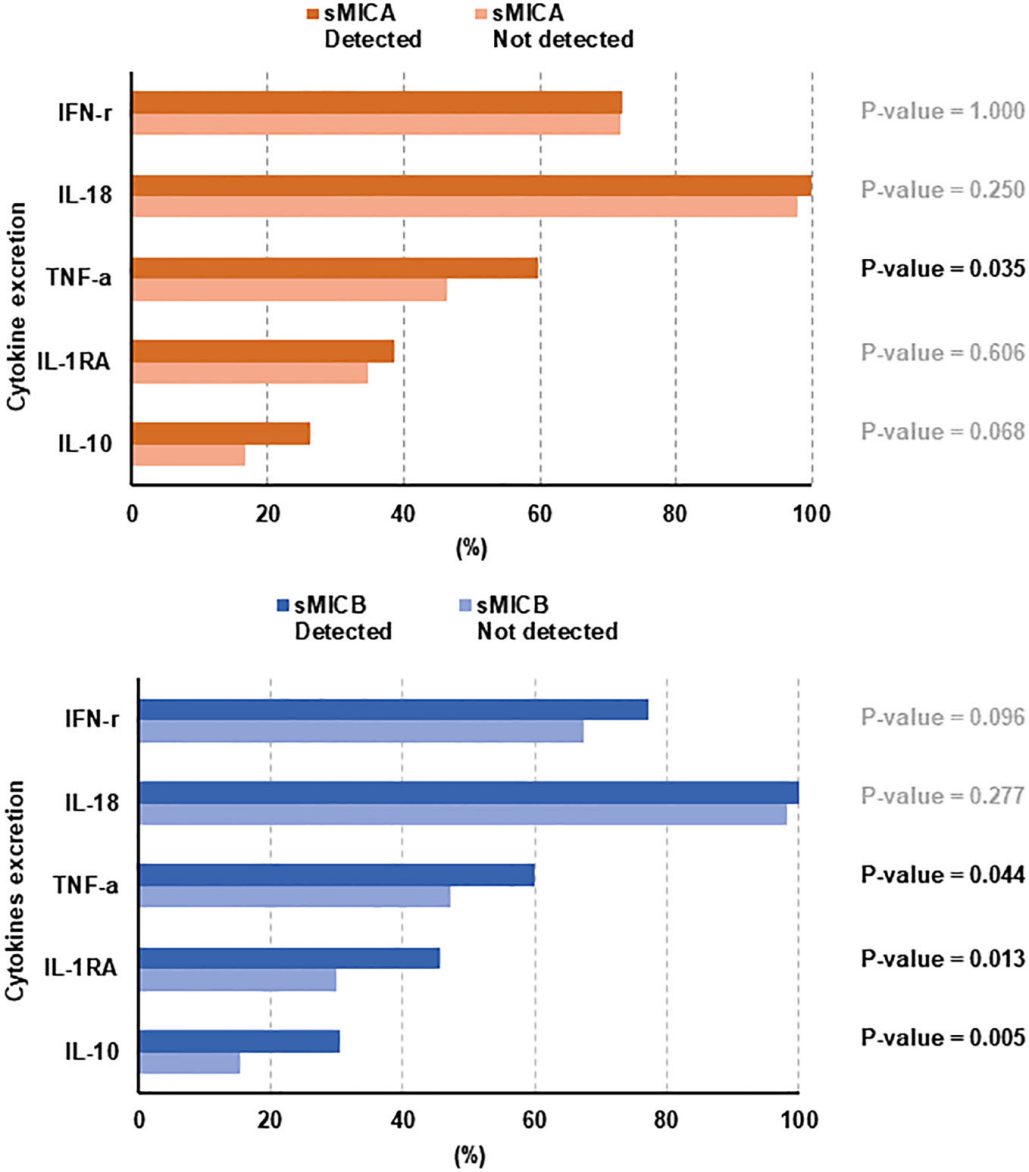
Correlation with the type of cytokine secretion according to sMICA/sMICB detection.

Of the 12 cytokines assessed, IL-18 (n = 259), IFN-γ (n = 190), TNF-α (n = 139), IL-1RA (n = 95), and IL-10 (n = 60) were detectable more frequently than IL-1β (n = 18), IL-12 (n = 8), IL-13(n = 8), IL-17α (n = 7), IL-6 (n = 7), IL-4 (n = 3), and IL-23 (n = 0). TNF-α, categorized as a pro-inflammatory cytokine, showed statistical significance in detecting sMICA (p = 0.035) or sMICB (p = 0.044). However, among anti-inflammatory cytokines, the presence of IL-1RA (p = 0.013) and IL-10 (p = 0.005) showed an association with the detection of sMICB, but they presented a weak correlation with the detection of sMICA (**Figure 4**). PFS in patients with the presence or higher detection of IL-1RA (p = 0.016), IL-10 (p = 0.011), and TNF-α (p = 0.019) was significantly worse than in those with no detection (**Supplemental Figure 3A**). The OS in patients with the detection of TNF-α (p = 0.016) was inferior to others (**Supplemental Figure 3B**). Furthermore, sMICA (p=0.313) or sMICB (p=0.695) detection was not correlated with CD68/CD163 ratio in tissue sample analysis (**Supplemental Figure 4**).

### Univariate and multivariate analysis of CNS involvement, PFS, and OS

In univariate analysis, elevated B2M (p < 0.001), elevated LDH (p<0.001), CNS involvement (p < 0.001), advanced-stage (p<0.001), high IPI (p < 0.001), detection of sMICA (p = 0.007), sMICB (p = 0.035), TNF-α (p = 0.021), IL-1RA (p = 0.018) and IL-10 (p = 0.013) presented a statistical correlation with inferior PFS. Among these variables, high B2M (p = 0.038), CNS involvement (p < 0.001), and advanced-stage (p=0.011) strongly correlated with poor PFS in the multivariate analysis. In univariate analysis of OS, elevated B2M (p = 0.003), elevated LDH (p<0.001), CNS involvement (p < 0.001), advanced-stage (p=0.001), high IPI (p = 0.001), and detection of TNF-α (p = 0.021) demonstrated significance, but only CNS involvement (p = 0.010) correlated with OS through multivariate analysis (**Table 2**).

**Table 2:** Cox regression analysis for estimating prediction markers of progression-free and overall survival.

Variables		Progression-free survival					
		Univariate analysis			Multivariate analysis		
		HR.	95% CI	*P*-value	HR.	95% CI	*P*-value
Gender	Male vs. Female	0.800	0.473-1.354	0.407			
B2M	Normal vs. Elevated	3.574	2.081-6.139	<0.001	1.965	1.039-3.718	0.038
LDH	Normal vs. Elevated	2.923	1.718-4.973	<0.001			
CNS involved	Involved vs. Not involved	11.843	6.873-20.406	<0.001	8.674	4.777-15.749	<0.001
Stage	I/II vs. III/IV	5.647	2.928-10.892	<0.001	3.436	1.333-8.858	0.011
IPI	<2 vs. ≥ 2	4.349	2.303-8.215	<0.001			
COO	GCB vs. non-GCB	0.875	0.605-1.267	0.480			
sMICA	Detection vs. Not detection	2.091	1.221-3.584	0.007			
sMICB	Detection vs. Not detection	1.746	1.041-2.926	0.035			
TNF-α	Detection vs. Not detection	1.892	1.101-3.252	0.021			
IL-1RA	Detection vs. Not detection	1.869	1.115-3.133	0.018			
IL-10	Detection vs. Not detection	2.030	1.161-3.548	0.013			
Variables		Overall survival					
		Univariate analysis			Multivariate analysis		
		HR	95% CI	*P*-value	HR	95% CI	*P*-value
Gender	Male vs. Female	0.834	0.387-1.798	0.644			
B2M	Normal vs. Elevated	3.499	1.530-7.998	0.003			
LDH	Normal vs. Elevated	5.661	2.284-14.032	<0.001			
CNS involved	Involved vs. Not involved	5.002	2.288-10.935	<0.001	3.067	1.301-7.231	0.010
Stage	I/II vs. III/IV	5.252	1.988-13.870	0.001			
IPI	<2 vs. ≥ 2	5.951	2.058-17.211	0.001			
COO	GCB vs. non-GCB	0.814	0.464-1.427	0.473			
sMICA	Detection vs. Not detection	1.825	0.828-4.021	0.136			
sMICB	Detection vs. Not detection	2.062	0.964-4.411	0.062			
TNF-α	Detection vs. Not detection	2.756	1.165-6.519	0.021			
IL-1RA	Detection vs. Not detection	1.511	0.706-3.232	0.287			
IL-10	Detection vs. Not detection	1.700	0.744-3.886	0.209			

sMICA, soluble major histocompatibility complex class I-related chain A; sMICB, soluble major histocompatibility complex class I-related chain B; P, P-value; ECOG, European Cooperative Oncology Group; PS, Performance status; LDH, Lactate dehydrogenase; COO, Cell-of-origin; IPI, International prognostic index.

## Discussion

Tumor immune evasion remains poorly understood because immune system formation is complex. Research on various solid cancers suggested that activating signaling between NKG2D receptors in NK cells and ligands (sMICA/sMICB) is involved in tumor immune response. Especially, sMICA, released by tumor cells, allows tumor cells to reduce NKG2D and NKG2D ligand surface expression and escape immune surveillance. sMICB is also reported to have a similar function to sMICA (2, 19, 20). Therefore, we performed a sample analysis study to establish the role of sMICA/sMICB, which interacts with the surrounding immune environment in ND-DLBCL. In the present study, the detection of sMICA/sMICB seemed to have a more critical relationship with inferior treatment outcomes. Moreover, ND-DLBCL patients with sMICA or sMICB might present lower overall response rate (ORR) and shorter PFS (**Figure 2**). In addition, an analytical correlation occurred between detecting sMICA or sMICB and cytokine excretion, such as IL-1RA, IL-10, and TNF-α, and the excretion of these cytokines correlated with the treatment outcomes (**Figure 4**; **Supplemental Figure 3**). However, the detection of sMICA, sMICB, or cytokines did not reflect the macrophage differentiation classified degree of expression or ratio of CD68/CD163 in tissue samples. Thus, sMICA or sMICB and various cytokines could be assumed to interact with each other and cooperate in orchestrating the tumor immune microenvironment to evade host immune surveillance, but the relationship with macrophage differentiation remains unclear.

NK cells, cytotoxic innate immune cells originating from lymphoid ancestors, are critical members in upfront anti-tumor activity and pro-inflammatory properties (21, 22). In an earlier study, the abundance of NK cells in head and neck squamous cell carcinoma had a better prognosis than minimal presentation (23). Moreover, a positive correlation between higher infiltration of NK cells and extended survival was reported in gastric and colorectal cancer (24, 25). A previous study reported that high levels of sMICA or sMICB might lead to the internalization and degradation of NKG2D receptors, resulting in the build-up of dysfunctional NK cells over time (26). In this study, we suggested the inferior survival outcomes of DLBCL patients who had sMICA or sMICB without reporting NK cell activity, but it could be hypothesized that sMICA or sMICB affects NK cell infiltration when previous studies are considered. Thus, sMICA or sMICB detection in ND-DLBCL seems to interrupt the immune balance against tumor clearance. The correlation between sMICA or sMICB expression and infiltration of NK cells in DLBCL tissue would need to be determined through further studies to definitively understand the role of sMICA or sMICB.

NKG2D ligands (MICA or MICB) are mainly induced at the cell surface by malignant evolution, rarely in healthy cells. Tumors expressing high levels of NKG2D ligands on cell surfaces showed extended survival in colorectal cancer, and high-level expression was observed frequently in limited-stage colorectal cancer (27). On the other hand, NKG2D expression and NK cell infiltration were shown less frequently in advanced-stage colorectal cancers, leading to a poor prognosis. It could be predicted that soluble NKG2D is present at higher levels in advanced stages. Consistent with these results, sMICA (P-value < 0.001) or sMICB (P-value = 0.019) were found at lower levels in limited-stage DLBCL than in advanced stages. Despite the same stage III or IV DLBCL, patients with the detection of sMICA or sMICB showed shorter PFS and OS (**Figure 3**). Therefore, the combination of sMICA or sMICB and the Ann Arbor stage seemed to have potential as a new predictor of survival outcomes. Considering its efficiency as a biomarker, measurement of NKG2D ligands in tissues could be performed in a limited number of patients with tissue samples, and results obtained through additional tissue sample management processes leading up to analysis. However, the assessment of sMICA or sMICB from serum could be undergone for all patients due to the convenient sample collection process, and results could be obtained without additional sample management processes. Nevertheless, it is still challenging to adjust the measurement of sMICA/sMICB for application in the clinic due to lack of validation, therefore more research is needed.

It is difficult to explain the mechanisms underlying sMICA/sMICB and cytokine contribution to tumor evasion due to their heterogeneity, which might reflect independent immune responses through different stress response pathways (28–30). In our study, we also analyzed cytokines by classifying them into two groups, pro-inflammatory (IFN-γ, IL-1β, IL-6, IL-12, IL-17α, IL-18, IL-23, and TNF-α) and anti-inflammatory (IL-1RA, IL-4, IL-10, and IL-13). The DLBCL patients with the detection of cytokines (IL1-RA, IL-10, and TNF-α) were observed to have shorter PFS. In this study, although we measured a specific cytokine excretion pattern in ND-DLBCL, demonstrating whether cytokines have a sincere correlation with sMICA or sMICB is still challenging due to the heterogeneous production and excretion of cytokines in DLBCL, which has unusual cell differentiation characteristics. Nevertheless, the increased cytokines in DLBCL could be considered a defense mechanism by which NK or cytotoxic T cells that are continuously exposed to sMICA or sMICB seek to establish host immune homeostasis through a self-regulatory mechanism (31).

Additionally, we analyzed the correlation between sMICA or sMICB expression and infiltration of macrophage cells in tumor tissues. However, we could not statistically demonstrate a correlation between sMICA (p = 0.313) or sMICB (p = 0.695) and tissue macrophage differentiation (**Supplemental Figure 4**). Some published studies evaluating TAMs in DLBCL have demonstrated that a higher CD68 or CD68/CD163 ratio was associated with M1 presentation and a favorable prognosis among DLBCL patients who received rituximab combined immunochemotherapy (14). However, other studies have emphasized that measuring the expression of CD68/CD163 lacked the evidence for implementation in the actual clinic as a predictive marker since macrophage is greatly affected by the surrounding environments. We also could not verify whether macrophage differentiation is influenced by sMICA or sMICB and cytokines, since the results do not reflect the entire immune environment, further research is needed to determine their relevance.

This represents the largest study to evaluate the significance of sMICA and sMICB on the survival of ND-DLBCL. Furthermore, only a few studies have assessed the levels of sMICA and sMICB and have not speculated on the correlation of the disease course. In the present study, sMICA or sMICB were related to the secretion of some cytokines, and the patients in who sMICA or sMICB were detected presented more advanced stages and shorter survival outcomes about immunochemotherapy. However, the limitation of this study is that 20.2% (n=53/262) reported the detection of sMICA/sMICB together due to the lack of a standardized assessment method that fully understands and reflects the distinct biology of sMICA/sMICB. Moreover, we did not present NK cell infiltration using CD56/CD16 staining and macrophage polarization through tissue sample analysis, so these results have a limitation in fully explaining the tumor microenvironment based on sMICA/sMICB. Thus, further study is needed to determine the role of sMICA/sMICB in the tumor microenvironment of DLBCL.

## Data availability statement

The original contributions presented in the study are included in the article/**Supplementary Material**, further inquiries can be directed to the corresponding author/s.

## Ethics statement

The studies involving humans were approved by the Samsung Medical Center IRB. The studies were conducted in accordance with the local legislation and institutional requirements. The participants provided their written informed consent to participate in this study.

## Author contributions

WSK and SEY conceived and designed the study. SEY, SP, JC, KJR, BY, SL, SJK, and WSK enrolled patients in the study and collected clinical data. SEY and SP analyzed the data and wrote the manuscript. All authors edited the manuscript and approved the final version of the manuscript.

## Supplementary material

Supplementary Figure 1 Cell segmentation and CD163-positive cell phenotyping based on tissue samples.

Supplementary Figure 2 Assessment of PFS and OF according to stages I/II and III/IV **(A)**, Comparison of median sMICA/sMICB levels between IPI 0-1 and IPI ≥ 2 **(B)**, Assessment of PFS and OF according to IPI 0-1 and IPI ≥ 2 **(C)**, Comparison of PFS and OS among four different categories: (1) the patients with IPI 0-1 and sMICA not detected, (2) those with IPI 0-1 and sMICA detection, (3) the patients with IPI ≥ 2 and sMICA not detected, and (4) those with IPI ≥ 2 and sMICA detection **(D)**, comparison of PFS and OS among four different categories: (1) the patients with IPI 0-1 and sMICB not detected, (2) those with IPI 0-1 and sMICB detection, (3) the patients with IPI ≥ 2 and sMICB not detected, and (4) those with IPI ≥ 2 and sMICB detection **(E)**.

Supplementary Figure 3 Comparison of PFS **(A)** and OS **(B)** according to IL-1RA, IL-10, and TNF-α detection.

Supplementary Figure 4 Comparison of log CD68/CD163 ratio according to sMICA or sMICB detection.
