# Supplementary figures and images for: The impact of sMICA/sMICB on immunochemotherapy outcomes in newly diagnosed diffuse large B-cell lymphoma
Source: Front Oncol. 2023 Nov 16;13:1194315. doi: 10.3389/fonc.2023.1194315 (PMC10687412; doi:10.3389/fonc.2023.1194315)

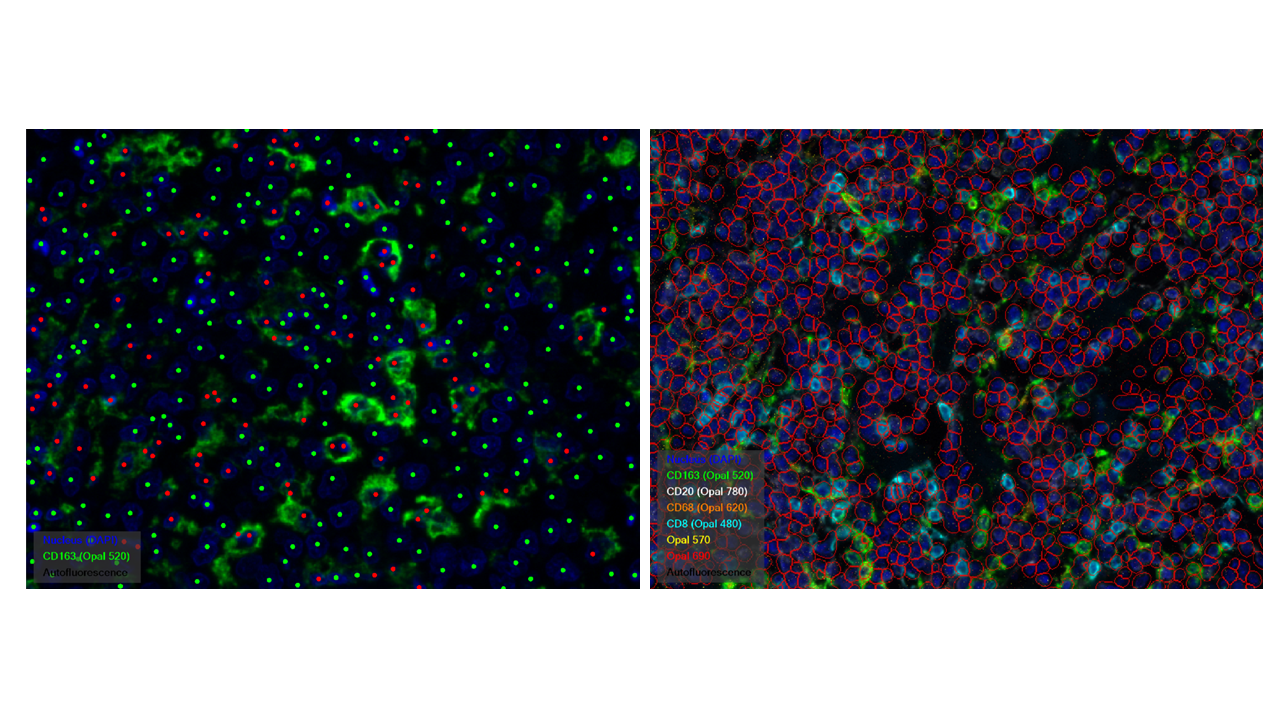

Supplement: Supplementary Figure 1 — Cell segmentation and CD163-positive cell phenotyping based on tissue samples. [file Image_1.tif]

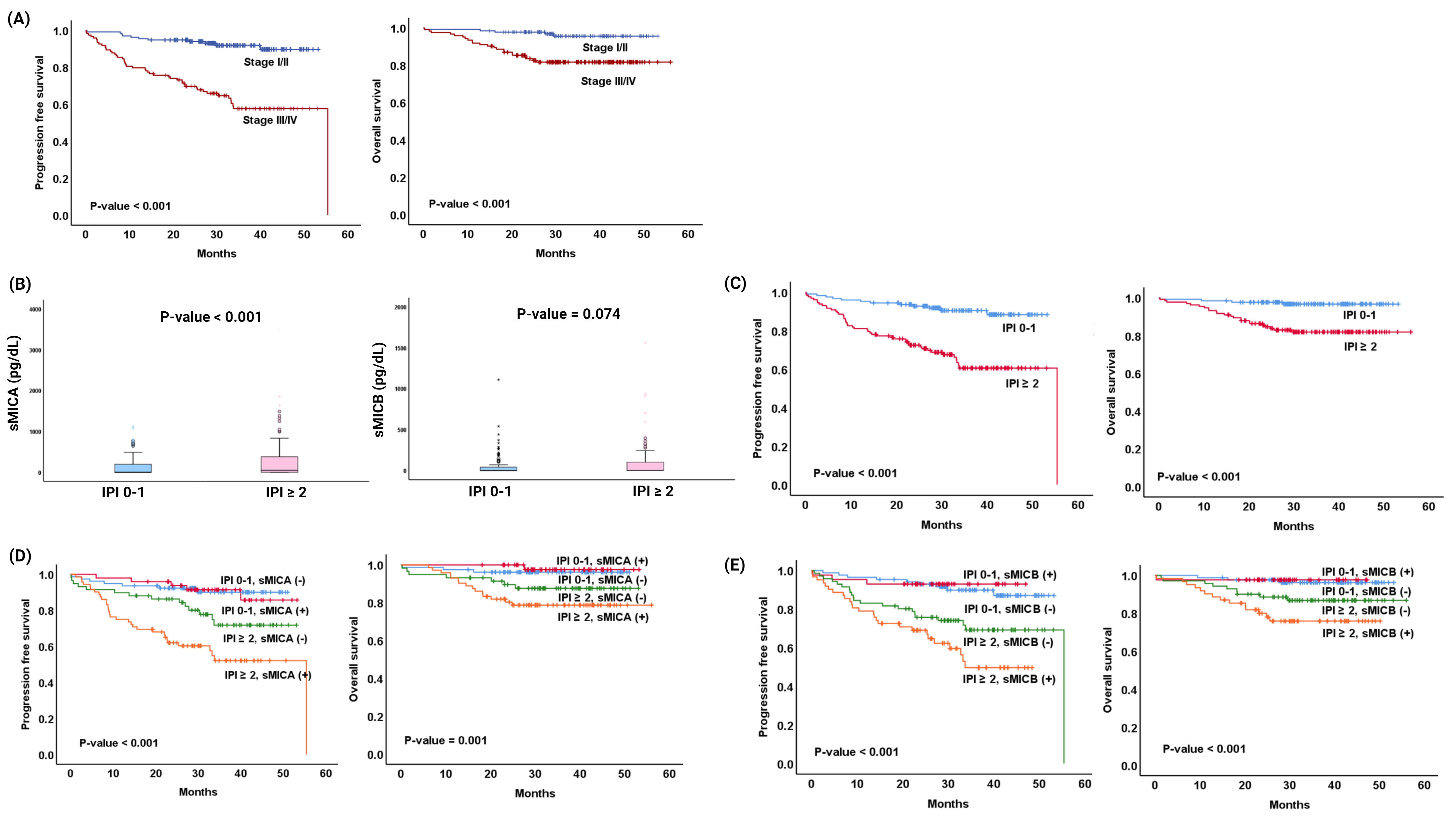

Supplement: Supplementary Figure 2 — Assessment of PFS and OF according to stages I/II and III/IV (A), Comparison of median sMICA/sMICB levels between IPI 0-1 and IPI ≥ 2 (B), Assessment of PFS and OF according to IPI 0-1 and IPI ≥ 2 (C), Comparison of PFS and OS among four different categories: (1) the patients with IPI 0-1 and sMICA not detected, (2) those with IPI 0-1 and sMICA detection, (3) the patients with IPI ≥ 2 and sMICA not detected, and (4) those with IPI ≥ 2 and sMICA detection (D), comparison of PFS and OS among four different categories: (1) the patients with IPI 0-1 and sMICB not detected, (2) those with IPI 0-1 and sMICB detection, (3) the patients with IPI ≥ 2 and sMICB not detected, and (4) those with IPI ≥ 2 and sMICB detection (E). [file Image_2.tif]

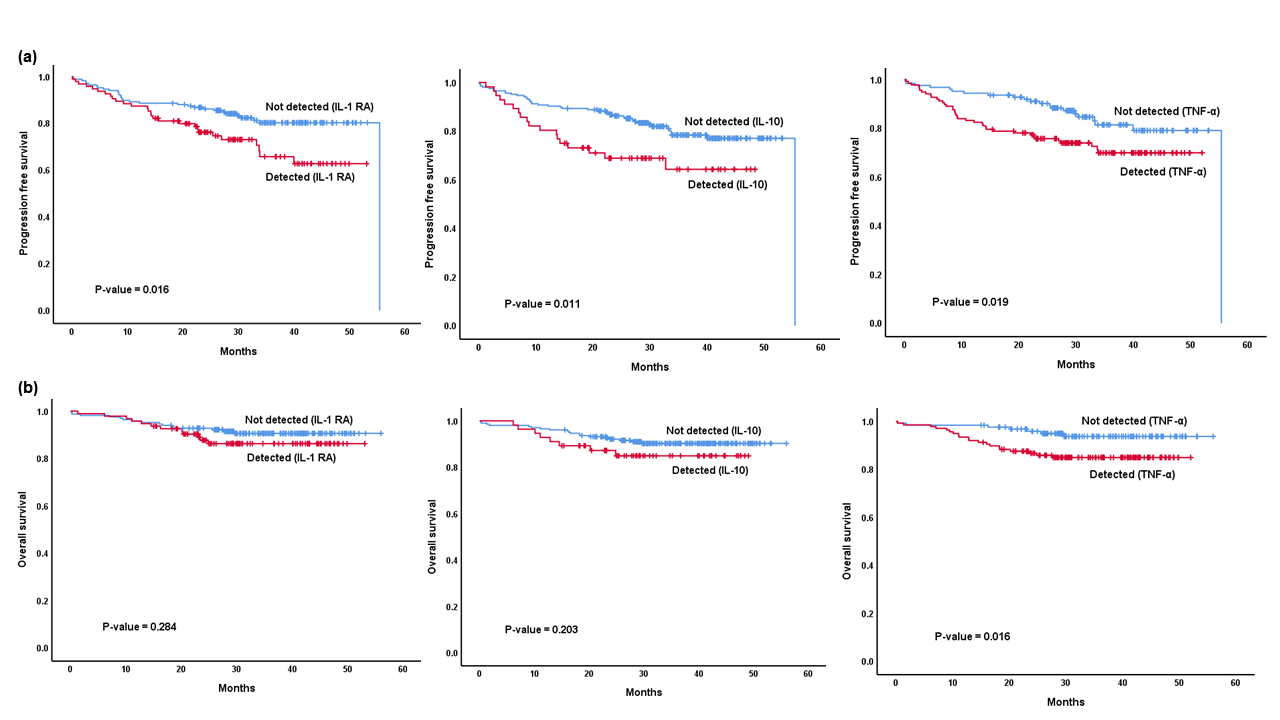

Supplement: Supplementary Figure 3 — Comparison of PFS (A) and OS (B) according to IL-1RA, IL-10, and TNF-α detection. [file Image_3.tif]

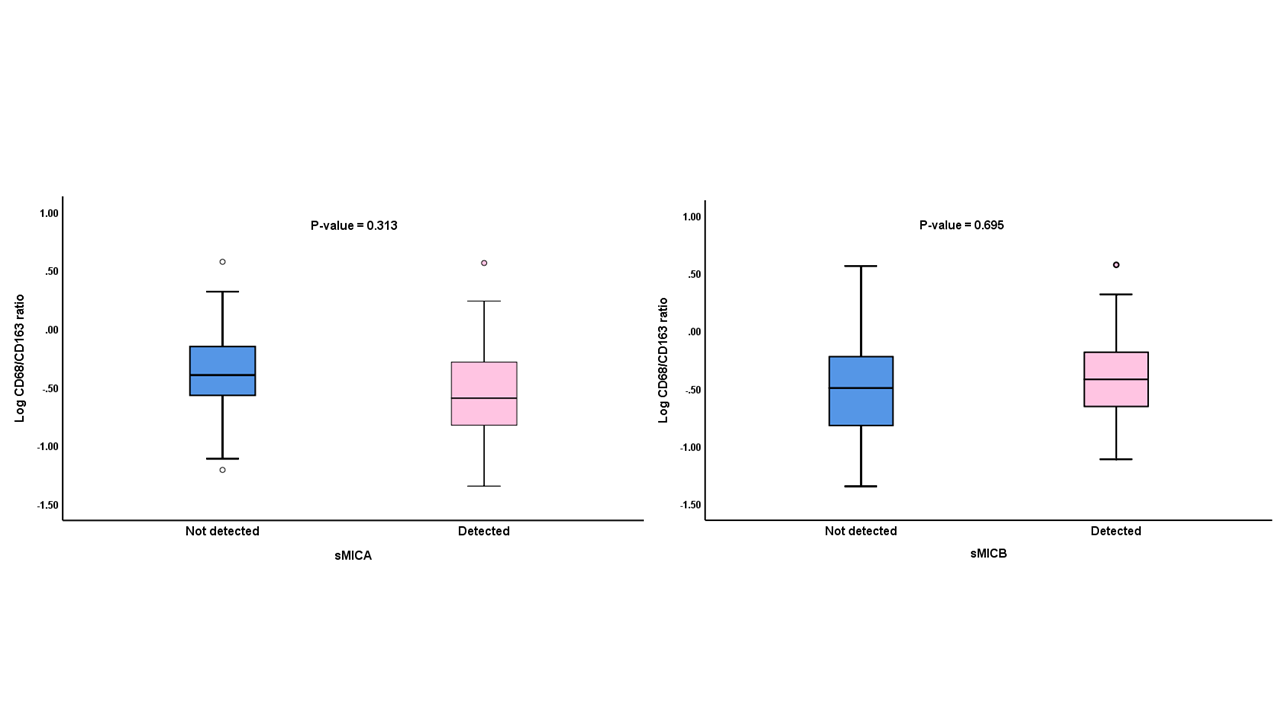

Supplement: Supplementary Figure 4 — Comparison of log CD68/CD163 ratio according to sMICA or sMICB detection. [file Image_4.tif]
